# The growing needs of genetic counselling—Feasibility in utilization of tele-genetic counselling in Asia and Hong Kong

**DOI:** 10.3389/fgene.2023.1239817

**Published:** 2023-08-03

**Authors:** Annie Tsz Wai Chu, Claudia Ching Yan Chung, Shirley Pik Ying Hue, Brian Hon Yin Chung

**Affiliations:** ^1^ Hong Kong Genome Institute, Hong Kong, Hong Kong SAR, China; ^2^ Department of Paediatrics and Adolescent Medicine, School of Clinical Medicine, Li Ka Shing Faculty of Medicine, The University of Hong Kong, Hong Kong, Hong Kong SAR, China

**Keywords:** tele-genetics, tele-genetic counselling, telemedicine, genetic counselling, Hong Kong Genome Project

## Abstract

The need for the expansion of genomic services has been at a record time high in the past decade. As technological advancement continues to strengthen the entire genetic and genomic pipeline and clinical operational workflow, the major challenge remains to be the speed of workforce development to meet service growth. In particular, the international expansion of genetic counselling (GC) services has been a topic of interest for the past few years. GC is an emerging profession in most of Asia, and in many countries the profession of GC often refers to physicians or front-line health workers with expertise in genetics to provide GC services rather than being a specific independent profession. As genetic and genomic services, especially pre-test and post-test GC, expand globally, the need to tackle the longstanding obstacles of GC personnel shortage and funding issues must not be overlooked. There is an urgent need internationally, and especially in Asia, where GC profession is comparatively less well-established, to seek alternative approaches to meet service demand. The present review examines the global development and feasibility of tele-genetics and tele-genetic counselling (TGC), and serves as the foundation to explore a possible roadmap in Hong Kong via the Hong Kong Genome Project.

## Introduction

In the past decade, telemedicine is being increasingly used in many areas of healthcare globally, particularly to improve cost efficiency and equitable access of health services in remote areas or for service-users experiencing difficulties in mobility, “in which interventions, diagnoses, therapeutic decisions, and subsequent treatment recommendations are based on patient data, documents and other information transmitted through telecommunication systems” (World Medical Association, 2022). With the increasing demand for clinical genetic services, lack of clinical geneticists and genetic counsellors, and the expertise of established genetic centers, telemedicine can be utilized in clinical genetics, an application that has been termed “tele-genetics” (Hilgart et al., 2012). It can serve as an effective method for GC and professional knowledge exchange. The coronavirus disease 2019 (COVID-19) pandemic has also catalyzed and accelerated the advancement and adoption of tele-genetics to minimize face-to-face interactions between healthcare providers and patients. This review aims to summarize the latest global evidence on tele-genetics and TGC, and to outline the background, current landscape, and aspirations of developing telemedicine and tele-genetics in Hong Kong China.

## Global development and acceptance of tele-genetics

Tele-genetics and TGC were introduced in various medical disciplines since the past two decades. The first application of TGC was formally reported in a study conducted in the United Kingdom (UK) in 1998, which was a pilot project in Wales that aimed to deliver TGC services in cancer genetics for patients living in geographically distant and underserved areas (Iredale et al., 2002). The United States (US), the leading pioneer in the development of the GC profession with the establishment of the US National Society of Genetic Counsellors (NSGC) in 1979, has also started practicing TGC in 2005 to increase accessibility to genetics education and clinical services in Maine (Lea et al., 2005). Previous studies evaluating the feasibility and cost-effectiveness of tele-genetics and TGC were typically conducted in North America and Europe, covering a wide range of genetic services in different areas, including but not limited to rare diseases and hereditary cancers. With majority of the genetic services being accessible in urban areas, especially during the initial stage of service development, tele-genetics and TGC as alternative methods were primarily initiated by Western countries to improve service accessibility among patients living in remote areas.

In recent years, advances in diagnostic testing of genetic conditions have led to an increased demand for genetic services globally. Growing application of tele-genetics has been introduced in multiple countries, including Canada, Germany, Italy, and the Netherlands, to ensure equitable access to services and to improve cost-efficiency. In addition to GC services, clinical genetic consultation including morphology assessment of pediatric patients was also shown to be acceptable by both patients and physicians via videoconferencing in an Australian study (Hopper et al., 2011), suggesting the feasibility and utility of tele-consultation and physical examination. However, many of the applications were in the pilot phase, implementation of tele-genetics in routine clinical practice would require standardization of workflows with stringent data privacy and security plans from expert panels to promote wider public acceptance and trust.

## Satisfaction level of tele-genetics: users vs. service providers

Tele-genetics and TGC can undoubtedly alleviate the problems of shortage of genetic counsellors, inaccessibility of GC service in remote areas, and inability of accessing in-person GC service among patients with mobility difficulties. However, it is important to evaluate their impact from the user’s and service provider’s perspective, including outcomes such as efficacy and satisfaction level. Table 1 summarizes patients’ and providers’ perspectives on tele-genetics and TGC. Regarding patients’ experiences in Western studies, the majority were satisfied with the quality of care from healthcare providers and reported significant knowledge gain post-TGC assessment. Genetic knowledge among patients was evaluated using an adapted six-item Cancer Genetics Knowledge scale in Bradbury et al.’s study of germline cancer genetics (Bradbury et al., 2018). Results indicated that patients who received TGC had a similar level of knowledge gain as compared to patients who received in-person GC, indicating TGC is reasonably a comparable alternative for knowledge exchange. Some studies also compared the economic and psychosocial outcomes from patients’ perspectives and reported TGC has no difference, or even preferable, to in-person GC. Chang et al. demonstrated a lower average cost of pre-test telephone GC ($120 USD) as compared to in-person GC ($270 USD) (Chang et al., 2016), providing evidence that TGC is beneficial for patients in saving costs for travelling, as well as saving time for accessing care (Jeganathan et al., 2020). In a study by Dratch et al., adult patients with neurological disorders declared a high level of satisfaction towards TGC, and expressed that their psychological concerns were alleviated after TGC, despite receiving a genetic diagnosis without available cure (Dratch et al., 2021). Bradbury et al. suggested that disclosure of genetic test results via telephone decreased patient’s state anxiety more than that of in-person disclosure (Bradbury et al., 2018). Interestingly, Farnè et al. have further compared the satisfaction levels of disclosing genetic test results via TGC in patients with different outcomes of genetic analysis (positive, negative, and variant of unknown significance (VUS)), and demonstrated that patients with VUS genetic test results have a greater interest for in-person GC, showing the need for them to be followed-up by the clinical geneticist or genetic counsellor face-to-face for the interpretation of their VUS variants, whose pathogenicity may change overtime (Farnè et al., 2023). Nevertheless, almost all patients expressed that they are willing to reuse TGC in the future (Farnè et al., 2023).

**TABLE 1:** Patients’ and providers’ perspective on tele-genetics and tele-genetic counselling.

Continent	Country	Setting	Patients’ perspective	Providers’ perspective
Asia (n = 7)	India (*n* = 2) Dawood et al. (2019); Rao et al. (2021)	Prenatal service and newborn screening: 29%	1. Patients were generally satisfied with the quality of services, but majority prefer in-person appointments for first and follow-up visits over telemedicine	1. Providers agreed that the use of telemedicine was a very useful tool for providing counselling during lockdown periods in 2020
	Israel (*n* = 1) Macarov et al. (2021)	Rare diseases: 29%	2. Patients were willing toward having genetic counselling by phone and video	
	Japan (*n* = 1) Nishiyama et al. (2021)	Hereditary cancers: 13%	3. Patients s’ age, employment status, income, mode of transportation to the appointment were associated with receptivity to TGC	
	Philippines (*n* = 1) Tumulak et al. (2021)	Others: 29%		
	Singapore (*n* = 2) Chin et al. (2020); Sim et al. (2021)			
Australia (n = 6)	Australia (*n* = 6) Gattas et al. (2001); Zilliacus et al. (2009); Zilliacus et al. (2010); Hopper et al. (2011); Zilliacus et al. (2011); Gonzalez et al. (2022)	Prenatal service and newborn screening: 0%	1. Patients reported generally high satisfaction levels in TGC	1. Providers were generally satisfied with tele-genetics and perceived high therapeutic alliance irrespective of appointment type
		Rare diseases: 17%		
		Hereditary cancers: 83%	2. Majority of patients agreed that tele-genetics was an effective way of seeing a geneticist for assessment	
		Others: 0%		
Europe (n = 9)	Germany (*n* = 1) Helmes et al. (2006)	Prenatal service and newborn screening: 11%	1. Majority of the patients are generally satisfied with the quality of tele-genetics and TGC	1. Providers’ trend of satisfaction level was lower with TGC than in-person GC
	Italy (*n* = 2) Pagliazzi et al. (2020); Farnè et al. (2023)	Rare diseases: 11%		
	Netherlands (*n* = 2) Otten et al. (2016a); Otten et al. (2016b)	Hereditary cancers: 67%		2. Providers felt less at ease and less able to observe patients’ nonverbal behavior
	Sweden (*n* = 2) (Platten et al. (2012); Pestoff et al. (2019))	Others: 11%	2. No differences in satisfaction level and psychosocial outcomes between the TGC and in-person GC groups	3. Providers identified three factors necessary for the implementation of TGC in clinical practice including the requirements for optimal use, the impact on clinical practice and the patients benefits
	United Kingdom (*n* = 2) Iredale et al. (2002); Coelho et al. (2005))			
North America (n = 43)	Canada (*n* = 5) (Burgess et al. (2016); Cremin et al. (2020); Hawkins et al. (2013); d'Agincourt-Canning et al. (2008); Sangha et al. (2003))	Prenatal service and newborn screening: 14%	1. Majority of the patients are generally satisfied with the quality of tele-genetics and TGC	1. Providers are concerned about technological limitations and difficulties in building rapport with patients in delivering GC services
	United States (*n* = 38) Lea et al. (2005); Abrams and Geier (2006); Stalker et al. (2006); Jenkins et al. (2007); Shanley et al. (2007); Baumanis et al. (2009); Sutphen et al. (2010); Bradbury et al. (2011); Meropol et al. (2011); Patrick-Miller et al. (2013); Patrick-Miller et al. (2014); Schwartz et al. (2014); Wenger et al. (2014); Buchanan et al. (2015); Butrick et al. (2015); Bradbury et al. (2016); Chang et al. (2016); Jacobs et al. (2016); Kinney et al. (2016); Mette et al. (2016); Peshkin et al. (2016); Cacioppo et al. (2017); Interrante et al. (2017); Steffen et al. (2017); Bradbury et al. (2018); Christensen et al. (2018); Fenton et al. (2018); Solomons et al. (2018); Voils et al. (2018); Zierhut et al. (2018); Terry et al. (2019); Arjunan et al. (2020); Gerrard et al. (2020); Greenberg et al. (2020); Jeganathan et al. (2020); Dratch et al. (2021); Liang et al. (2022); Falah et al. (2023)	Rare diseases: 7%	2. No differences in psychosocial outcomes and genetic knowledge gain between the TGC and in-person GC groups	2. Telemedicine aids in improving providers’ workflow and reducing costs for outpatients’ services
		Hereditary cancers: 67%	3. Patients who received TGC had a similar level of knowledge gain as compared to patients who received in-person GC, indicating TGC is reasonably a comparable alternative for knowledge exchange	
		Others: 12%	4. Patients were willing to consider using telemedicine for future visits if it was appropriately adopted	

The successful implementation of any service depends on the service provider’s perception, expectations, and satisfaction level, which are determining factors for the long-term sustenance of service. Although high satisfaction level among patients utilizing tele-genetics or TGC services are validated by various patient-centric studies, few studies have incorporated the examination of expectations and satisfaction level from the healthcare provider’s perspective. In Australia, tele-genetics has been demonstrated to yield high satisfaction level among practitioners. Practitioners perceived that TGC offers several benefits including the improvement of service efficiency, the ability to adopt flexible working patterns such as working from home, and the reduction of travelling costs and time for outpatient service (Zilliacus et al., 2009; Gonzalez et al., 2022).

Despite gaining positive feedback from service users and providers, concerns have been raised by service providers in maintaining high quality tele-genetics or TGC services, which have caused variable levels of satisfaction among providers. A pilot study from the Netherlands reported experiences and concerns among genetic counsellors in online counselling sessions. Results particularly demonstrated a lower overall mean Telemedicine Satisfaction Questionnaire (TSQ) (Yip et al., 2003) score among genetic counsellors after online counselling session as compared to their baseline expectations (Otten et al., 2016a). In Australia, resolving technical issues, coordinating genetic testing remotely and managing patient work were also reported to have made tele-genetics or TGC more time-consuming as compared to in-person GC, which lowered the level of provider’s satisfaction (Gonzalez et al., 2022). Building rapport with patients, especially in assessing and detecting nonverbal cues and emotions can be difficult at times and are perceived as major hiccups for online counselling in the study by (Otten et al., 2016b). Nevertheless, this has not been observed as an obstacle to tele-genetics and TGC in a more recent study by Gonzalez et al. (Gonzalez et al., 2022). Genetic counsellors were satisfied with tele-genetics or TGC using videoconferencing and perceived high therapeutic alliance irrespective of appointment type, suggesting tele-genetics or TGC does not comprise the genetic counsellors’ ability to convey empathy for rapport building with patients. Besides, there is no difference in time assessment between tele-genetics and standard care including in-person and outreach telephone GC.

While tele-genetics or TGC is a comparable alternative method for delivering clinical genetics and GC services, strategic planning and other supports in building and strengthening therapeutic alliance during the GC sessions are also the indispensable prerequisites. For instance, training sessions can be provided to service providers to familiarize them with telecommunication tools and equipment to increase their confidence in adopting GC via virtual platforms. In addition, guidelines should be developed for assessing patients’ body language to minimize the chance of missing patients’ non-verbal cues and psychosocial needs.

## Tele-genetics development in Asia

The development and adoption of tele-genetics is relatively slow in Asia in comparison to the West. Pilot studies have been conducted in India, Israel, Japan, Singapore, and the Philippines. The majority of these studies were published during the period of the COVID-19 pandemic to minimize face-to-face interaction and thus the risk of transmission between patients and providers. In Singapore, Sim et al. conducted a pilot study among 160 patients to assess their views and willingness towards the use of telemedicine in a cancer genetics service in 2019 and illustrated that most of the patients were willing to have genetic services by phone (71.3%) and videoconferencing (66.3%) (Sim et al., 2021). They were also satisfied with the quality of the services and felt comfortable when sharing information on the virtual platform. However, they preferred in-person GC for first (79.4%) and TGC for follow-up (60.6%) visits rather than telemedicine as an entire alternative model. A study from India also reported that 87% of the patients were satisfied with TGC services, but 74% of them preferred face-to-face appointment (Rao et al., 2021).

In general, patients from Asian countries such as Singapore and Japan, have a relatively lower preference for telemedicine models in comparison with patients from Western countries such as the US and Australia, even if they have confidence in the quality of care from healthcare providers. Potential factors and reasons have been suggested in multiple studies. For instance, cultural perception of confidence is instilled by meeting healthcare providers in person (Sim et al., 2021). They might be under the impression that it will be more difficult to build a connection with the healthcare providers via telemedicine, particularly verbalizing their psychosocial needs. Asian populations may be more conservative with the new approach and require time for adaptation. Unlike large countries such as the US where patients may need to travel to another city for health services, smaller regions like Hong Kong and Singapore have genetic services that are more reachable and accessible, usually within two to three hours of total commute time. Therefore, geographical constraints may not be the reason for utilizing tele-genetics services in smaller countries. Moreover, genetic and genomic services, including genetic screening, genetic health promotion, and GC, are still emerging professions with limited labor force and institutional providers in Asia (Dawood et al., 2019). As such, tele-genetics has not been widely implemented in the Asian healthcare systems when compared to Western countries. However, considering the impetus of the COVID-19 pandemic globally where social distancing is restricted, telemedicine and tele-genetics are alternative options for accessing care.

Participants’ age, socioeconomic status, health, and technological literacy were also found to be associated with receptivity to tele-genetics in a few studies. In the US, Buchanan et al. and Patrick-Miller et al. illustrated that non-attenders of TGC are mainly non-white, are more likely to have a household income of less than $50,000 USD, and are generally less comfortable with technology (Patrick-Miller et al., 2013; Buchanan et al., 2015). In Japan, Nishiyama et al. conducted a study on pregnant women’s opinions toward prenatal GC in different models (Nishiyama et al., 2021). While majority of the women preferred in-person GC rather than TGC, their preferences varied between different background characteristics, experiences, attitudes, and health literacy. Future studies should take account into patient’s characteristics, as well as appointment types and disease groups.

The above overview summarizes global development trends and outcomes of tele-genetics and TGC. Although tele-genetics has been widely utilized in some Western countries such as the US to improve cost-efficiency and equitable access, it has not been widely adopted in the context of the Asia-pacific region due to various reasons. Possible practical constraints and challenges include the lack of resources and professionals (Nishiyama et al., 2021). Consent and data confidentiality on the virtual platform is also a global growing concern. Supportive regulations and guidelines are needed for fostering the acceptance of tele-genetics in routine clinical praxis.

## Background and current landscape of developing telemedicine and tele-genetics in Hong Kong

Since Hong Kong is a geographically small and densely populated city with well-established commute infrastructures, public hospitals and clinics are divided into designated district zones. Citizens can reach their designated public hospital within an hour from their living places. As such, the development of telemedicine and tele-genetic services has traditionally been slow in Hong Kong. To improve service accessibility for patients with mobility difficulties, the Hospital Authority (HA) has started to use videoconference to replace conventional face-to-face psychiatric services such as geriatric outreach and follow-up consultations for elderly patients who live in residential homes since 1998 (Cheng, 2021). The positive feedback and advantages have driven HA to extend the telemedicine network and establish the Community Health Call Centre in two hospitals to support not only elderly patients but also patients with chronic disease by telephone consultations (Hui et al., 2001; Hui, 2008; Chan et al., 2011; Kwong, 2018). The COVID-19 pandemic has further accelerated the adoption of telemedicine and tele-genetics in Hong Kong to minimize face-to-face interactions between healthcare providers and patients. As technological advancement provides solutions to escalating service demand, the “Smart Hospital Project” has been implemented in the 2019–20 Policy Address during the COVID-19 pandemic, coupling with the hospital navigation app “HA GO” considering its convenience and high accessibility nature (Food and Health Bureau, 2019; Cheng, 2021). Patients can make use of the online functions such as appointment booking and medication enquiry to facilitate hospital service efficiency.

The usage of telemedicine has also been growing in the private sector including private hospitals and clinics. For example, the Hong Kong Sanatorium Hospital has set up teleconsultation services in 2020 to cater for the needs of patients for outpatient services during the COVID-19 pandemic (Hong Kong Sanatorium and Hospital, 2020). On the other hand, “DrGo” provides telemedicine services through a mobile app partnering with serval private clinics such as Quality Healthcare and EC Healthcare (DrGo, 2023). In order to give proper guidance to medical practitioners taking part in telemedicine services, the Hong Kong Medical Council published the “Ethical Guidelines on Practice of Telemedicine in December 2019” (Medical Council of Hong Kong, 2019). In one of these guidelines, medical practitioners have been reminded to remain fully responsible for meeting all legal and ethical requirements when practicing telemedicine. Besides, doctors should align with the principles and standards guiding privacy, security of records, and informed consent. Since the ethical guidelines have not been clearly defined for specific conditions and procedures, doctors are cautious in adopting telemedicine services, which hinders broad adoption of telemedicine in Hong Kong as of to date. As such, ethical and practice guidelines should be formulated in different specialties to facilitate routine clinical application of telemedicine.

Although telemedicine has been successfully implemented in Hong Kong in various medical specialties in recent years, it has been lagging in its usage in clinical genetic services, including GC. Recently in 2022, telemedicine has been used in a genetic consultation with patient’s parents due to travel restrictions during the COVID-19 pandemic in Hong Kong. This is a piece of evidence that the practice of telemedicine can touch upon the clinical genetic services in Hong Kong, demonstrating the feasibility of implementing tele-genetics in health systems.

## Aspirations for future development in Hong Kong: Hong Kong Genome Project

As evidenced in the above, there is a trend in developing tele-genetics and TGC worldwide, with generally high satisfaction from the perspectives of patients and providers. According to the 2022 Professional Status Survey by the NSGC, tele-genetics (audio-visual) has become the most common service delivery model in 2021 in North America (82%), followed by in-person GC (81%) and telephone consultation (74%) (National Society of Genetic Counselors, 2022). While the driving force of development and perceived benefits of tele-genetics and TGC varied among places, it is commonly agreed that TGC can help to solve the problems of shortage of genetic counsellors.

With the perceived benefits of tele-genetics and TGC, large-scale genomic sequencing projects have also utilized tele-genetics and TGC in routine clinical care and for research purposes. For instance, Geisinger’s precision health project, MyCode, a health system-based biobanking program, has also integrated tele-genetics and TGC into their genetic research and counselling centers since 2015 (Bresnick, 2015; Carey et al., 2016). Telecommunication tools, such as videoconferencing, allows researchers, clinicians and patients in the Geisinger Health System to share their views and opinions on the impact of genetics on the development and treatment of diseases without geographical constraints. During the pandemic of the COVID-19, Geisinger expanded its telehealth services to include primary care and over 70 specialties including genetic and genomic services to provide the participants with continued access to health services. Another genomics research program, the NYCKidSeq, has launched the TeleKidSeq pilot study recently to use telemedicine as an alternative form of genomic service delivery model for participants from medically underserved populations to undergo genome sequencing (Sebastin et al., 2023). This study aims to evaluate the impact of TGC in communicating genome sequencing results to the participants, and assess participant outcomes including understanding, satisfaction, psychological and socio-economic implications. These large-scale projects provide evidence in the feasibility of integrating telemedicine in clinical genetics, which serves as the basis for health systems around the world to plan for future development of tele-genetics and TGC.

GC is an emerging profession in Hong Kong. It is estimated that Hong Kong currently has less than 15 full-time personnels practicing GC services in the public healthcare system. GC services led by the clinical geneticists, have been provided at the Clinical Genetic Service (CGS) of Department of Health in three genetic counselling clinics since 1981, where the patients or families are assessed and provided with appropriate genetic testing to substantiate the genetic diagnosis. CGS handles about 1,800 new cases every year, with a cumulative caseload involving more than 43,000 families (up to 2020) since its establishment. As genetic and genomic services expand, the number of genetic counsellors is still limited. With a profession still in its establishing stage, there are few reasons why face-to-face GC should remain as the major service delivery model in Hong Kong. Firstly, the general population is not familiar with GC and its scope and standard of practice. Traditional face-to-face GC facilitates the building of professional image of genetic counsellors and their understanding of GC profession. Secondly, face-to face GC allows the building of therapeutic alliance between the genetic counsellor and the patient/family members. Thirdly, newly trained genetic counsellor may also benefit from face-to-face GC sessions through observation and direct supervision by an experienced genetic counsellor and clinical geneticist.

Since there are limited genetic counsellors in Hong Kong, it is common for senior genetic counsellors and clinical geneticists to attend multiple hospitals/clinics to reach their patients or to provide supervision and trainings. Although geographical constraint may not be the major reason for using tele-genetics in Hong Kong as patients can access healthcare services within a few hours of commute, day-to-day travelling time of genetic counsellors between centers constitute a significant hindrance in service efficiency. A hybrid mode of GC service may be a feasible and sustainable option in the long run. The first GC session can be face-to-face to facilitate the building of therapeutic alliance, while for subsequent or follow-up sessions patients can be given the options to receive GC services face-to-face or via telemedicine. For result-disclosure, service providers may opt for TGC for disclosing negative results.

The commencement of the Hong Kong Genome Project, the first large-scale whole-genome sequencing project in Hong Kong, acts as a catalyst for the development of genomic medicine and provides a platform for developing GC profession (Chu et al., 2022; Chu et al., 2023). This creates an opportunity in developing and building necessary infrastructure in enhancing service provision to translate genetic and genomic research into clinical practices. The use of telemedicine in genetics and GC services is helpful in supporting service provision by improving the efficiency of the workforce, particularly in whole-genome sequencing result disclosure and post-test GC.

Although the development of tele-genetics and TGC service in Hong Kong is still at a conceptual stage, the current implementation of telemedicine in public and private healthcare sectors demonstrates its potential in clinical application. Along with this trend of implementation, the formulation of ethical, data security, and practice guidelines, standardization of workflows, and fostering of public engagement are needed to facilitate the process.

## Conclusion and future directions

This review provides a summary on the latest global development and feasibility of tele-genetics and TGC, and serves as the foundation to explore a possible roadmap in Hong Kong. Learning from the experience from other large-scale genomic sequencing projects worldwide such as the Geisinger’s precision health project and the TeleKidSeq pilot study, it sheds light on the possibility of integrating telemedicine into clinical genetics in Hong Kong, such as via the Hong Kong Genome Project. With the large-scale of patient recruits, and a team of trained genetic counsellors dedicating full-time in this project, the Hong Kong Genome Project can be a driving pioneer engaging important stakeholders in the industry to research, design, test, and implement a sustainable model of TGC in the foreseeable future.

## References

[B1] Abrams D. J. Geier M. R. (2006). A comparison of patient satisfaction with telehealth and on-site consultations A pilot study for prenatal genetic counseling. J. Genet. Couns. Jun 15 (3). 199–205. 10.1007/s10897-006-9020-0 16779676

[B2] Arjunan A. Ben-Shachar R. Kostialik J. Johansen Taber K. Lazarin G. A. Denne E. (2020). Technology-riven oninvasive renatal creening esults isclosure and anagement. Telemed. J. E Health. Jan. 26 (1), 8–17. 10.1089/tmj.2018.0253 PMC6948005 30807262

[B3] Baumanis L. Evans J. P. Callanan N. Susswein L. R. (2009). Telephoned BRCA1/2 genetic test results: Revalence, practice, and patient satisfaction. J. Genet. Couns. Oct. 18 (5), 447–463. 10.1007/s10897-009-9238-8 19462222

[B4] Bradbury A. Patrick-Miller L. Harris D. Stevens E. Egleston B. Smith K. (2016). Utilizing emote eal-ime ideoconferencing to xpand ccess to ancer enetic ervices in ommunity ractices: A ulticenter easibility tudy. J. Med. Internet Res. 1 18 (2), e23. 10.2196/jmir.4564 PMC4754531 26831751

[B5] Bradbury A. R. Patrick-Miller L. Fetzer D. Egleston B. Cummings S. A. Forman A. (2011). Genetic counselor opinions of, and experiences with telephone communication of BRCA1/2 test results. Clin. Genet. 79 (2), 125–131. 10.1111/j.1399-0004.2010.01540.x 21039431 PMC3059740

[B6] Bradbury A. R. Patrick-Miller L. J. Egleston B. L. Hall M. J. Domchek S. M. Daly M. B. (2018). Randomized oninferiority rial of elephone vs n-erson isclosure of ermline ancer enetic est esults. J. Natl. Cancer Inst. 110 (9), 985–993. 10.1093/jnci/djy015 29490071 PMC6136932

[B7] Bresnick J. (2015). New geisinger health center merges telehealth. *Genomics* [Online]. Available at: https://mhealthintelligence.com/news/new-geisinger-health-center-merges-telehealth-genomics .

[B8] Buchanan A. H. Datta S. K. Skinner C. S. Hollowell G. P. Beresford H. F. Freeland T. (2015). Randomized rial of elegenetics vs. In-erson ancer enetic ounseling: Cost, atient atisfaction and ttendance. J. Genet. Couns. 24 (6), 961–970. 10.1007/s10897-015-9836-6 25833335 PMC4592683

[B9] Burgess K. R. Carmany E. P. Trepanier A. M. (2016). A omparison of elephone enetic ounseling and n-erson enetic ounseling from the enetic ounselor's erspective. J. Genet. Couns. 25 (1), 112–126. 10.1007/s10897-015-9848-2 26044544

[B10] Butrick M. Kelly S. Peshkin B. N. Luta G. Nusbaum R. Hooker G. W. (2015). Disparities in uptake of BRCA1/2 genetic testing in a randomized trial of telephone counseling. Genet. Med. 17 (6), 467–475. 10.1038/gim.2014.125 25232856 PMC4364924

[B11] Cacioppo C. Reddy N. Wood E. M. Jaeger J. Ofidis D. Sawhney R. (2017). Improving ccess to ancer enetic ounseling through elegenetics - ayhealth - niversity of Pennsylvania nitiative. J. Public Health 3 (3), 10–13. 10.32481/djph.2017.06.004 PMC8396628 34466913

[B12] Carey D. J. Fetterolf S. N. Davis F. D. Faucett W. A. Kirchner H. L. Mirshahi U. (2016). The geisinger MyCode community health initiative: An electronic health record-linked biobank for precision medicine research. Genet. Med. 18, 906–913. 10.1038/gim.2015.187 26866580 PMC4981567

[B13] Chan F. W. Wong F. Y. Fung H. Yeoh E. K. (2011). The development of a Health Call Centre in Hong Kong: A study on the perceived needs of patients. Hong Kong Med. J. 17, 208–216. 21636869

[B14] Chang Y. Near A. M. Butler K. M. Hoeffken A. Edwards S. L. Stroup A. M. (2016). Economic evaluation Alongside a clinical trial of telephone versus in-person genetic counseling for BRCA1/2 Mutations in Geographically underserved Areas. J. Oncol. Pract, 12 (1), e1-e13. 10.1200/JOP.2015.004838 PMC4960460 26759468

[B15] Cheng I. (2021). Development of telehealth services [online]. Lesgislative Council, the Hong Kong special administrative region of the people's Republic of China. Available: https://www.legco.gov.hk/research-publications/english/essentials-2021ise14-development-of-telehealth-services.htm .

[B16] Chin X. W. Ang Z. L. T. Tan R. Y. C. Courtney E. Shaw T. Chen Y. (2020). Use of telephone intake for family history taking at a cancer genetics service in Asia. J. Genet. Couns. 29 (6), 1192–1199. 10.1002/jgc4.1286 32368849

[B17] Christensen K. D. Uhlmann W. R. Roberts J. S. Linnenbringer E. Whitehouse P. J. Royal C. D. M. (2018). A randomized controlled trial of disclosing genetic risk information for Alzheimer disease via telephone. Genet. Med. Jan. 20 (1), 132–141. 10.1038/gim.2017.103 PMC5897910 28726810

[B18] Chu A. T. W. Chung C. C. Y. Hong Kong Genome P. Lo S. Chung B. H. Y. (2023). Marketing and publicity strategies for launching the pilot phase of the Hong Kong Genome Project. J. Transl. Genet. Genomics 7, 66–78. 10.20517/jtgg.2023.01

[B19] Chu A. T. W. Fung J. L. F. Tong A. H. Y. Chow S. M. Chan K. Y. K. Yeung K. S. (2022). Potentials and challenges of launching the pilot phase of Hong Kong Genome Project. J. Transl. Genet. Genomics 6, 290–303. 10.20517/jtgg.2022.02

[B20] Coelho J. J. Arnold A. Nayler J. Tischkowitz M. MacKay J. (2005). An assessment of the efficacy of cancer genetic counselling using real-time videoconferencing technology (telemedicine) compared to face-to-face consultations. Eur. J. Cancer 41 (15), 2257–2261. 10.1016/j.ejca.2005.06.020 16176873

[B21] Cremin C. Lee M. K. Hong Q. Hoeschen C. Mackenzie A. Dixon K. (2020). Burden of hereditary cancer susceptibility in unselected patients with pancreatic ductal adenocarcinoma referred for germline screening. Cancer Med. 9 (11), 4004–4013. 10.1002/cam4.2973 32255556 PMC7286471

[B22] d'Agincourt-Canning L. McGillivray B. Panabaker K. Scott J. Pearn A. Ridge Y. (2008). Evaluation of genetic counseling for hereditary cancer by videoconference in British Columbia. B Med. J. 50, 554–559.

[B23] Dawood S. S. Dhar A. Sharma P. Kini L. (2019). Development of a telegenetics program in the Asia-Pacific/Middle East region. J. Glob. Oncol. 5 (l), 15. 10.1200/JGO.2019.5.suppl.15

[B24] Dratch L. Paul R. A. Baldwin A. Brzozowski M. Gonzalez-Alegre P. Tropea T. F. (2021). Transitioning to telegenetics in the COVID-19 era: Patient satisfaction with remote genetic counseling in adult neurology. J. Genet. Couns. 30 (4), 974–983. 10.1002/jgc4.1470 34265143 PMC8427091

[B25] DRGO (2023). DrGo - video consultation with a doctor on the go [Online]. Available at: https://www.drgo.com.hk/index_en.html .

[B26] Falah N. Terry A. Umer A. Kastner M. Oliverio K. L. Matthews N. (2023). A pilot study of home-based genetic testing completion rate in telegenetics cancer clinics in West Virginia Appalachia. Am. J. Med. Genet. A. Jan. 191, 1013–1019. 10.1002/ajmg.a.63109 36637370

[B27] Farnè M. Fortunato F. Neri M. Farnè M. Balla C. Albamonte E. (2023). TeleNEwCARe: An Italian case-control telegenetics study in patients with Hereditary NEuromuscular and CARdiac diseases. Eur. J. Med. Genet. 66, 104749. 10.1016/j.ejmg.2023.104749 36948289

[B28] Fenton G. L. Smit A. K. Freeman L. Badcock C. Dunlop K. Butow P. N. (2018). Development and valuation of a elephone ommunication rotocol for the elivery of ersonalized elanoma enomic isk to the eneral opulation. J. Genet. Couns. 27 (2), 370–380. 10.1007/s10897-017-0183-7 29199389

[B29] Food And Health Bureau (2019). “LC paper No. CB(2)13/19-20(01 legislative Council panel on health services, 2019 policy address, policy initiatives of the food and health Bureau,” in The Hong Kong special administrative region of the PEOPLE'S Republic of China.

[B30] Gattas M. R. MacMillan J. C. Meinecke I. Loane M. Wootton R. (2001). Telemedicine and clinical genetics: Stablishing a successful service. J. Telemed. 7 (Suppl. 2), 68–70. 10.1258/1357633011937191 11747665

[B31] Gerrard S. Inglis A. Morris E. Austin J. (2020). Relationships between patient- and session-related variables and outcomes of psychiatric genetic counseling. Eur. J. Hum. Genet. 28 (7), 907–914. 10.1038/s41431-020-0592-1 32066934 PMC7316807

[B32] Gonzalez T. K. Tucker C. E. Wakefield P. Geelan-Small P. Macmillan S. Taylor N. (2022). Comparing cancer genetic counselling using telegenetics with in-person and telephone appointments: Results of a partially randomised patient-preference pilot study. J. Telemed., 1357633X2211125. 10.1177/1357633x221112556 35833346

[B33] Greenberg S. E. Boothe E. Delaney C. L. Noss R. Cohen S. A. (2020). Genetic ounseling ervice elivery odels in the United States: Assessment of changes in use from 2010 to 2017. J. Genet. Couns. 29 (6), 1126–1141. 10.1002/jgc4.1265 32314856

[B34] Hawkins A. K. Creighton S. Ho A. McManus B. Hayden M. R. (2013). Providing predictive testing for untington disease via telehealth: Esults of a pilot study in British olumbia, Canada. Clin. Genet. 84 (1), 60–64. 10.1111/cge.12033 23039041

[B35] Helmes A. W. Culver J. O. Bowen D. J. (2006). Results of a randomized study of telephone versus in-person breast cancer risk counseling. Patient Educ. Couns. 64 (1-3), 96–103. 10.1016/j.pec.2005.12.002 16427245

[B36] Hilgart J. S. Hayward J. A. Coles B. Iredale R. (2012). Telegenetics: A systematic review of telemedicine in genetics services. Genet. Med. 14, 765–776. 10.1038/gim.2012.40 22498847

[B37] HONG KONG SANATORIUM & HOSPITAL (2020). Telemedicine service [online]. Available at: https://mobile.hksh.com/en/whats-new/hksh-telemedicine-service-launched .

[B38] Hopper B. Buckman M. Edwards M. (2011). Evaluation of satisfaction of parents with the use of videoconferencing for a pediatric genetic consultation. Res. Hum. Genet. 14 (4), 343–346. 10.1375/twin.14.4.343 21787118

[B39] Hui E. (2008). Telemedicine & tele-rehabilitation in elderly care. Hong Kong: HA Convention.

[B40] Hui E. Woo J. Hjelm M. Zhang Y. T. Tsui H. T. (2001). Telemedicine: A pilot study in nursing home residents. Gerontology 47, 82–87. 10.1159/000052778 11287732

[B41] Interrante M. K. Segal H. Peshkin B. N. Valdimarsdottir H. B. Nusbaum R. Similuk M. (2017). Randomized oninferiority rial of elephone vs n-erson enetic ounseling for ereditary reast and varian ancer: A 12-onth ollow-p. Cancer Spectr. 1 (1), pkx002. 10.1093/jncics/pkx002 PMC6611491 31304457

[B42] Iredale R. Gray J. Murtagh G. (2002). Telegenetics: A ilot tudy of ideo-ediated enetic onsultations in ales. J. Med. Mark. 2 (2), 130–135. 10.1057/palgrave.jmm.5040066

[B43] Jacobs A. S. Schwartz M. D. Valdimarsdottir H. Nusbaum R. H. Hooker G. W. DeMarco T. A. (2016). Patient and genetic counselor perceptions of in-person versus telephone genetic counseling for hereditary breast/ovarian cancer. Fam. Cancer 15 (4), 529–539. 10.1007/s10689-016-9900-x 26969308 PMC5011450

[B44] Jeganathan S. Prasannan L. Blitz M. J. Vohra N. Rochelson B. Meirowitz N. (2020). Adherence and acceptability of telehealth appointments for high-risk obstetrical patients during the coronavirus disease 2019 pandemic. Am. J. Obstet. Gynecol. Nov. 2 (4), 100233. 10.1016/j.ajogmf.2020.100233 PMC7506329 32984803

[B45] Jenkins J. Calzone K. A. Dimond E. Liewehr D. J. Steinberg S. M. Jourkiv O. (2007). Randomized comparison of phone versus in-person BRCA1/2 predisposition genetic test result disclosure counseling. Genet. Med. 9 (8), 487–495. 10.1097/gim.0b013e31812e6220 17700386

[B46] Kinney A. Y. Steffen L. E. Brumbach B. H. Kohlmann W. Du R. Lee J. H. (2016). Randomized oninferiority rial of elephone elivery of BRCA1/2 enetic ounseling ompared ith n-erson ounseling: 1-Year ollow-p. J. Clin. Oncol. 34 (24), 2914–2924. 10.1200/JCO.2015.65.9557 27325848 PMC5012661

[B47] Kwong S. Y. P. (2018). Patient support Call Centre: A territory-wide structured telephone support for HA patients 13th hkec symposium on community engagement 2018 - paradigm shift: From hospital to community 2018 Hong Kong hospital authority.

[B48] Lea D. H. Johnson J. L. Ellingwood S. Allan W. Patel A. Smith R. (2005). Telegenetics in Maine: Successful clinical and educational service delivery model developed from a 3-year pilot project. Genet. Med. Jan. 7 (1), 21–27. 10.1097/01.gim.0000151150.20570.e7 15654224

[B49] Liang L. W. Kalia I. Latif F. Waase M. P. Shimada Y. J. Sayer G. (2022). The use of telemedicine in cardiogenetics clinical practice during the COVID-19 pandemic. Mol. Genet. Med 10, e1946. 10.1002/mgg3.1946 PMC9184656 35388985

[B50] Macarov M. Schneider N. Eilat A. Yahalom C. (2021). Genetic counseling practice for inherited eye diseases in an Israeli medical center during the COVID-19 pandemic. J. Genet. Couns. 30 (4), 969–973. 10.1002/jgc4.1479 34378273 PMC8426981

[B51] MEDICAL COUNCIL OF HONG KONG (2019). Ethical guidelines on practice of telemedicine online. Available at: https://www.mchk.org.hk/files/PDF_File_Ethical_Guidelines_on_Telemedicine.pdf .

[B52] Meropol N. J. Daly M. B. Vig H. S. Manion F. J. Manne S. L. Mazar C. (2011). Delivery of nternet-based cancer genetic counselling services to patients' homes: feasibility study. J. Telemed. 17 (1), 36–40. 10.1258/jtt.2010.100116 PMC3263376 21097566

[B53] Mette L. A. Saldivar A. M. Poullard N. E. Torres I. C. Seth S. G. Pollock B. H. (2016). Reaching high-risk underserved individuals for cancer genetic counseling by video-teleconferencing. J. Community Support Oncol. 14 (4), 162–168. 10.12788/jcso.0247 27152515

[B54] NATIONAL SOCIETY OF GENETIC COUNSELORS (2022). 2022 professional status Survey - executive summary online. Available at: https://www.nsgc.org/Portals/0/Executive%20Summary%20Final%2005-03-22.pdf .

[B55] Nishiyama M. Ogawa K. Hasegawa F. Sekido Y. Sasaki A. Akaishi R. (2021). Pregnant women's opinions toward prenatal pretest genetic counseling in Japan. J. Hum. Genet. 66 (7), 659–669. 10.1038/s10038-021-00902-4 33486503 PMC7825380

[B56] Otten E. Birnie E. Ranchor A. V. van Langen I. M. (2016a). Online genetic counseling from the providers' perspective: Ounselors' evaluations and a time and cost analysis. Eur. J. Hum. Genet. 24 (9), 1255–1261. 10.1038/ejhg.2015.283 26785833 PMC4989197

[B57] Otten E. Birnie E. Ranchor A. V. van Langen I. M. (2016b). Telegenetics use in presymptomatic genetic counselling: Atient evaluations on satisfaction and quality of care. Eur. J. Hum. Genet. 24 (4), 513–520. 10.1038/ejhg.2015.164 26173963 PMC4929881

[B58] Pagliazzi A. Mancano G. Forzano G. di Giovanni F. Gori G. Traficante G. (2020). Genetic counseling during COVID-19 pandemic: Tuscany experience. Mol. Genet. Med 8 (10), e1433. 10.1002/mgg3.1433 PMC7435534 32743952

[B59] Patrick-Miller L. Egleston B. L. Daly M. Stevens E. Fetzer D. Forman A. (2013). Implementation and outcomes of telephone disclosure of clinical BRCA1/2 test results. Patient Educ. Couns. 93 (3), 413–419. 10.1016/j.pec.2013.08.009 24075727 PMC4199583

[B60] Patrick-Miller L. J. Egleston B. L. Fetzer D. Forman A. Bealin L. Rybak C. (2014). Development of a communication protocol for telephone disclosure of genetic test results for cancer predisposition. Res. Protoc. 3 (4), e49. 10.2196/resprot.3337 PMC4259920 25355401

[B61] Peshkin B. N. Kelly S. Nusbaum R. H. Similuk M. DeMarco T. A. Hooker G. W. (2016). Patient erceptions of elephone vs. In-erson BRCA1/BRCA2 enetic ounseling. J. Genet. Couns. 25 (3), 472–482. 10.1007/s10897-015-9897-6 26455498 PMC4829475

[B62] Pestoff R. Johansson P. Nilsen P. Gunnarsson C. (2019). Factors influencing use of telegenetic counseling: Erceptions of health care professionals in Sweden. J. Community Genet. 10 (3), 407–415. 10.1007/s12687-018-00404-5 30617812 PMC6591505

[B63] Platten U. Rantala J. Lindblom A. Brandberg Y. Lindgren G. Arver B. (2012). The use of telephone in genetic counseling versus in-person counseling: randomized study on counselees' outcome. Fam. Cancer 11 (3), 371–379. 10.1007/s10689-012-9522-x 22399327 PMC3496516

[B64] Rao N. Kanago D. Morris M. Narayan V. Varshney K. Gn S. (2021). Telegenetics: The experience of an Indian center (Centre for uman enetics) during the COVID-19 pandemic. J. Genet. Couns. 30 (5), 1224–1232. 10.1002/jgc4.1517 34596296 PMC8657350

[B65] Sangha K. K. Dircks A. Langlois S. (2003). Assessment of the ffectiveness of enetic ounseling by elephone ompared to a linic isit. J. Genet. Couns. 12 (2), 171–184. 10.1023/A:1022663324006 26140847

[B66] Schwartz M. D. Valdimarsdottir H. B. Peshkin B. N. Mandelblatt J. Nusbaum R. Huang A. T. (2014). Randomized noninferiority trial of telephone versus in-person genetic counseling for hereditary breast and ovarian cancer. J. Clin. Oncol. Mar. 1 32 (7), 618–626. 10.1200/JCO.2013.51.3226 PMC3927731 24449235

[B67] Sebastin M. Odgis J. A. Suckiel S. A. Bonini K. E. Di Biase M. Brown K. (2023). The TeleKidSeq pilot study: Incorporating telehealth into clinical care of children from diverse backgrounds undergoing whole genome sequencing. Pilot Feasibility Stud. 9, 47. 10.1186/s40814-023-01259-5 36949526 PMC10031704

[B68] Shanley S. Myhill K. Doherty R. Ardern-Jones A. Hall S. Vince C. (2007). Delivery of cancer genetics services: The Royal Marsden telephone clinic model. Fam. Cancer 6 (2), 213–219. 10.1007/s10689-007-9131-2 17508269

[B69] Sim J. Shaw T. Li S. T. Courtney E. Yuen J. Chiang J. (2021). Understanding patients' views and willingness toward the use of telehealth in a cancer genetics service in Asia. J. Genet. Couns. 30 (6), 1658–1670. 10.1002/jgc4.1432 33934420

[B70] Solomons N. M. Lamb A. E. Lucas F. L. McDonald E. F. Miesfeldt S. (2018). Examination of the atient-ocused mpact of ancer elegenetics mong a ural opulation: Comparison with raditional n-erson ervices. Telemed. J. E Health. 24 (2), 130–138. 10.1089/tmj.2017.0073 28737998 PMC8259038

[B71] Stalker H. J. Wilson R. McCune H. Gonzalez J. Moffett M. Zori R. T. (2006). Telegenetic medicine: Mproved access to services in an underserved area. J. Telemed. 12 (4), 182–185. 10.1258/135763306777488762 16774698

[B72] Steffen L. E. Du R. Gammon A. Mandelblatt J. S. Kohlmann W. K. Lee J. H. (2017). Genetic esting in a opulation-ased ample of reast and varian ancer urvivors from the REACH andomized rial: Cost arriers and oderators of ounseling ode. Cancer Epidemiol. Prev 26 (12), 1772–1780. 10.1158/1055-9965.EPI-17-0389 PMC5712253 28971986

[B73] Sutphen R. Davila B. Shappell H. Holtje T. Vadaparampil S. Friedman S. (2010). Real world experience with cancer genetic counseling via telephone. Fam. Cancer 9 (4), 681–689. 10.1007/s10689-010-9369-y 20799063 PMC3303219

[B74] Terry A. B. Wylie A. Raspa M. Vogel B. Sanghavi K. Djurdjinovic L. (2019). Clinical models of telehealth in genetics: A regional telegenetics landscape. J. Genet. Couns. 28 (3), 673–691. 10.1002/jgc4.1088 30825358

[B75] Tumulak M. J. R. Pascua A. V. Jover E. J. M. Guerbo R. J. Canoy G. M. R. Laurino M. Y. (2021). Genetic counseling in the time of COVID-19: The Philippine experience with telegenetics. J. Genet. Couns. 30 (5), 1285–1291. 10.1002/jgc4.1518 34558759 PMC8657528

[B76] Voils C. I. Venne V. L. Weidenbacher H. Sperber N. Datta S. (2018). Comparison of elephone and elevideo odes for elivery of enetic ounseling: andomized rial. J. Genet. Couns. 27 (2), 339–348. 10.1007/s10897-017-0189-1 29243007

[B77] Wenger T. L. Gerdes J. Taub K. Swarr D. T. Deardorff M. A. Abend N. S. (2014). Telemedicine for genetic and neurologic evaluation in the neonatal intensive care unit. J. Perinatol. Mar. 34 (3), 234–240. 10.1038/jp.2013.159 PMC3943754 24406740

[B78] WORLD MEDICAL ASSOCIATION (2022). Archived: WMA statement on the ethics of telemedicine [online]. Available at: https://www.wma.net/policies-post/wma-statement-on-the-ethics-of-telemedicine/ .

[B79] Yip M. P. Chang A. M. Chan J. Mackenzie A. E. (2003). Development of the telemedicine satisfaction Questionnaire to evaluate patient satisfaction with telemedicine: A preliminary study. J. Telemed. Telecare 9, 46–50. 10.1258/135763303321159693 12641893

[B80] Zierhut H. A. MacFarlane I. M. Ahmed Z. Davies J. (2018). Genetic ounselors' xperiences and nterest in elegenetics and emote ounseling. J. Genet. Couns. 27 (2), 329–338. 10.1007/s10897-017-0200-x 29362948

[B81] Zilliacus E. Meiser B. Lobb E. Barlow-Stewart K. Tucker K. (2009). A balancing act--telehealth cancer genetics and practitioners' experiences of a triadic consultation. J. Genet. Couns. 18 (6), 598–605. 10.1007/s10897-009-9247-7 19798555

[B82] Zilliacus E. Meiser B. Lobb E. Dudding T. E. Barlow-Stewart K. Tucker K. (2010). The virtual consultation: Ractitioners' experiences of genetic counseling by videoconferencing in Australia. Telemed. J. E Health 16 (3), 350–357. 10.1089/tmj.2009.0108 20406122

[B83] Zilliacus E. M. Meiser B. Lobb E. A. Kelly P. J. Barlow-Stewart K. Kirk J. A. (2011). Are videoconferenced consultations as effective as face-to-face consultations for hereditary breast and ovarian cancer genetic counseling? Genet. Med. Nov. 13 (11), 933–941. 10.1097/GIM.0b013e3182217a19 21799430

